# P-1041. Contemporary Increase in Candidemia Mortality Is Associated with Antifungal Resistance and Patient Demographics

**DOI:** 10.1093/ofid/ofae631.1231

**Published:** 2025-01-29

**Authors:** Max W Adelman, Masayuki Nigo, Ashton Connor, Stefano Casarin, Enshuo Hsu, Cesar A Arias

**Affiliations:** Houston Methodist Hospital, Houston, Texas; Houston Methodist Hospital, Houston, Texas; Houston Methodist Hospital, Houston, Texas; Houston Methodist Hospital, Houston, Texas; Houston Methodist Hospital, Houston, Texas; Houston Methodist and Weill Cornell Medical College, Houston, TX

## Abstract

**Background:**

*Candida* spp. commonly cause bloodstream infections (BSI) in chronically ill patients, and *Candida* BSI is increasingly due to antifungal-resistant species. This retrospective cohort study examined temporal trends in candidemia epidemiology and associations between epidemiologic changes and mortality.

**Table 1.**
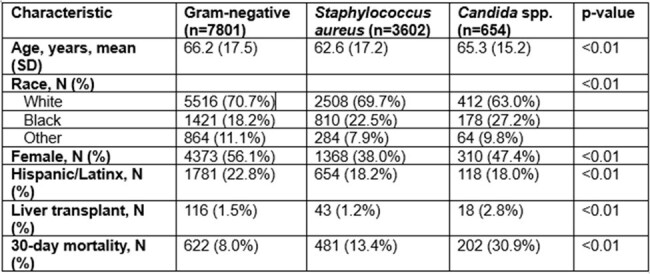
Characteristics of patients with different bloodstream infection types.

**Methods:**

We included patients in a large healthcare system with a first BSI due to *Escherichia coli* or *Klebsiella pneumonia* (gram-negative bacteremia [GNB]), *Staphylococcus aureus* (SAB), or *Candida* spp. (candidemia) from 6/2016-6/2023. We compared 30-day mortality and demographics between BSI types. We examined changes in candidemia demographics, mortality, and causative species, both by individual year and by period (period 1, 2016-2019 vs. period 2, 2020-2023).

**Figure 1.**
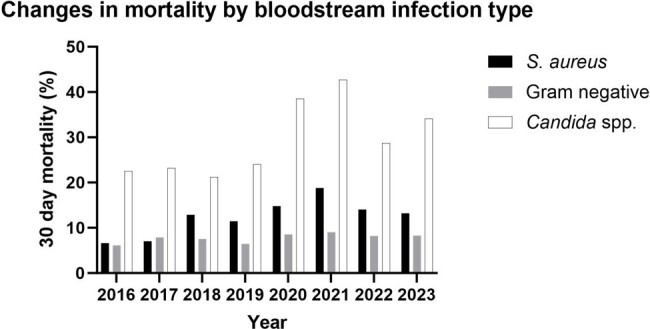
Changes in mortality due to bloodstream infection types examined.

**Results:**

Of 12057 patients, 7801 (64.7%) had GNB, 3602 (29.9%) SAB, and 654 (5.4%) candidemia (baseline characteristics in **Table 1**). Thirty-day mortality was highest for candidemia (30.9% vs. 13.4% for SAB and 8.0% for GNB, p< .01 by χ^2^). There was a trend toward increased mortality for patients with candidemia (p=0.06 by Mann-Kendall test, **Figure 1**). Candidemia mortality was significantly higher in period 2 vs. 1 (36.8% vs. 22.7%, p< .01 by χ^2^). Candidemia patients in period 2 tended to be older (mean age 66.3±14.3 vs. 63.8±16.2, p=0.04 by *t-*test); age was associated with mortality among patients with candidemia (OR 1.02, 95% CI 1.01-1.04 per year increase). Of species examined, only the incidence of *C. auris* increased (p< 0.01 by Mann-Kendall test, **Figure 2**). *C. glabrata* was associated with the highest 30-day mortality (36.3%) among common species; *C. auris* mortality was 17.4%. While the proportion of candidemia cases due to antifungal-resistant species (*C. auris*, *glabrata*, *krusei*, *lusitaniae*, *parapsilosis*) did not change between periods (p=0.79), mortality from these species was significantly higher in period 2 vs. 1 (36.1% vs. 21.5%, p< 0.01).

**Figure 2.**
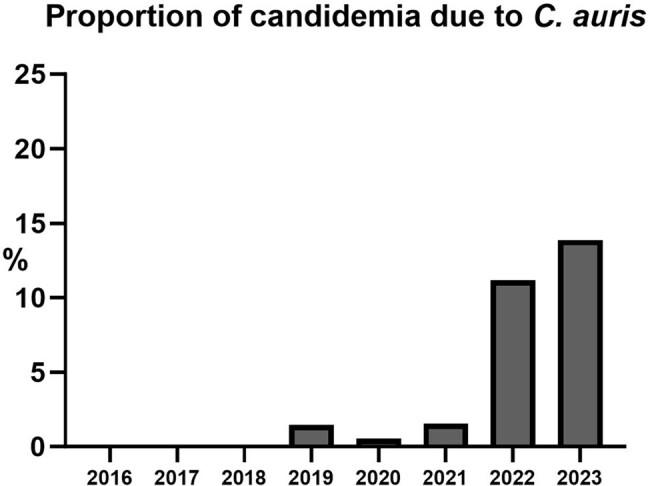
Increasing prevalence of candidemia due to C. auris, 2016-2023.

**Conclusion:**

Candidemia mortality increased significantly starting in 2020, at least in part due to increasing patient age and increased mortality associated with antifungal-resistant candidemia. Urgent efforts are needed to combat candidemia in an ageing population prone to antifungal-resistant *Candida* infections.

**Disclosures:**

**All Authors**: No reported disclosures

